# Clinical Outcome Differences in Mucinous Versus Non-Mucinous Colonic Adenocarcinoma: A Comparative Study

**DOI:** 10.3390/diagnostics15020192

**Published:** 2025-01-15

**Authors:** Adrian Cote, Roxana Loriana Negrut, Hany Abdulateif Salem, Bogdan Feder, Mircea Gheorghe Pop, Adrian Marius Maghiar

**Affiliations:** 1 County Clinical Emergency Hospital Bihor, 410087 Oradea, Romania; adrian.cote@didactic.uoradea.ro (A.C.); bogdanfeder@yahoo.com (B.F.); pgmircea@gmail.com (M.G.P.); 2 Department of Surgical Disciplines, Faculty of Medicine and Pharmacy, University of Oradea, 410073 Oradea, Romania; 3 Department of Medicine, Doctoral School of Biomedical Sciences, Faculty of Medicine and Pharmacy, University of Oradea, 410087 Oradea, Romania; 4 Egyptian Ministry of Health and Population, Cairo 71529, Egypt

**Keywords:** colon cancer, mucinous adenocarcinoma, colon malignancy, histopathological types of colon cancer

## Abstract

**Background/Objectives**: Colon cancer is one of the main causes of cancer-related mortality worldwide. Among its histopathological subtypes, mucinous adenocarcinoma (MAC) is characterized by a more aggressive behavior than non-mucinous adenocarcinoma (non-MAC). This study aimed to compare the clinical outcomes and postoperative recovery between MAC and non-MAC cases in order to better understand the treatment implications and optimize therapeutic strategies. **Methods**: A retrospective cohort study was conducted on patients diagnosed and treated at the Bihor County Emergency Hospital between January 2019 and December 2022. Data were collected from the medical records. Patients were divided into two groups, based on the histopathological results: mucinous adenocarcinoma and non-mucinous adenocarcinoma. Statistical analysis included descriptive statistics, *t*-tests, Chi-square tests, and ANOVA where appropriate. **Results**: A total of 191 patients were enrolled in this study, grouped in 36 cases of MAC and 155 cases of non-MAC. No significant statistical differences were found regarding hematological parameters. However, MAC was associated with higher rates of local invasion and a predominant right-sided colonic location, necessitating more frequent right colectomies. The overall mortality rate was significantly higher for MAC, indicating its aggressive nature. **Conclusions**: MAC presents higher local invasion rates and overall mortality. The aggressiveness of MAC underscores the need for tailored treatment approaches to optimize patient outcomes. Future large-scale studies are recommended to validate these findings and refine the therapeutic strategies.

## 1. Introduction

Colon cancer ranks fourth in global incidence and fifth in cancer-related mortality, with an estimated 1,142,286 diagnosed cases reported by the World Health Organization and Global Cancer Observatory in 2022 [1]. Colon mucinous adenocarcinoma (C-MAC) is a distinct subtype of cancer, which is characterized by its high content of extracellular mucin, a jelly-like substance, which constitutes over 50% of the tumor mass [2]. Evidence derived from the literature proved that colorectal mucinous adenocarcinoma (CR-MAC) accounts for nearly 4% to 19% of all colorectal cancers, with a prevalence ranging from 10% to 15% worldwide [3,4].

While the incidence of CRC, including MAC, has increased steeply in many parts of the world [5], the incidence demonstrated a significant reduction in other regions. For instance, in the United States, the rate of CR-MAC has seen a decline from 4.5 per 100,000 persons per year in 2000 to 1.54 per 100,000 persons per year in 2018 [1,3]. These varying trends may be attributed to differences in screening programs, lifestyle factors, and access to healthcare. In contrast, Romania lacks a national screening program, which may contribute to higher undiagnosed cases.

This rare subtype has garnered significant attention for its distinct epidemiological and clinical features, including differences in demographic patterns, tumor progression, and prognosis compared to non-mucinous colorectal cancers [4]. A significant research gap still exists regarding the detailed understanding of these differences and their implications in treatment.

Current evidence from the literature indicates that MAC tends to have worse overall survival (OS) and disease-free survival (DFS) rates. A survival analysis for colon MAC revealed that the 5-year DFS was 67% following surgery and the 5-year survival probability of patients who survived 4 years or more was 98%; the 5-year OS was 73% after surgery and increased to 92% after surviving four years post-surgery [6]. These findings highlight the need for different treatment strategies for MAC to improve clinical outcomes.

In Romania, the age-standardized cancer rate is estimated to be 276.5 per 100,000 [7], with nearly 95,000 new cases of CRC diagnosed in 2020 [8,9]. Moreover, the World Health Organization reported that CRC ranks among the top ten causes of mortality in Romania, regardless of age or sex [8,10]. Notwithstanding, the specific epidemiological biostatistics for adenocarcinoma are hardly separated by subtype. The lack of differentiation in epidemiological statistics highlights the need for better recognition of MAC in clinical and research settings [11].

We hypothesize that mucinous adenocarcinoma exhibits distinct clinical and laboratory outcomes compared to non-mucinous adenocarcinoma.

Despite the growing interest in investigating the CRC, the MAC phenotype remains under-represented in decision-making guidelines, potentially leading to inconsistent management and prognosis [12,13]. Therefore, to address this gap, this retrospective cohort study aims to characterize the postoperative clinical outcomes of patients diagnosed with mucinous adenocarcinoma of the colon. By evaluating tumor-related factors, surgical approaches, length of hospital stay, morbidity, and mortality, this study seeks to inform evidence-based surgical practices and optimize patient outcomes.

## 2. Materials and Methods

A retrospective cohort study was conducted to compare clinical and postoperative outcomes in patients diagnosed with colon cancer who underwent surgery at a single surgical department within the Bihor County Emergency Hospital between January 2019 and December 2022. Based on the histopathologic results, this study focused on comparing the patients diagnosed with mucinous adenocarcinoma to those with non-mucinous tumors.

### 2.1. Inclusion and Exclusion Criteria

Patients were continuously included over the study period, based on the inclusion and exclusion criteria, without any intervention to ensure equal group size. This approach preserves the integrity of the cohort by accurately representing the natural distribution of these subtypes.

Adults aged 18 or older with a histologically confirmed diagnosis of colon malignancy were included in this study, regardless of whether they presented as emergency or elective cases. Emergency cases included those presenting in the Emergency Department with bowel obstruction, peritonitis, hemorrhage, or sepsis due to tumor-related complications such as abdominal wall abscess. Elective cases were defined as patients undergoing planned surgeries without acute complications that require immediate intervention.

Exclusion criteria comprised individuals with a history of malignancy within the previous 5 years and those experiencing tumor recurrence.

### 2.2. Data Collection

For the research, information was gathered from medical records. This involved obtaining demographic details (age and gender), as well as tumor characteristics (tumor location, TNM stage, grading, size, lymphovascular and perineural invasion, and node involvement), surgical details (type of surgery performed, nature of anastomosis, and timing from admission to surgery). In addition, postoperative data were documented including any immediate complications, the occurrence and timing of the fistula, length of hospital stay, and survival during hospitalization. Furthermore, hematological data included information about white blood cell count (WBC), lymphocyte count, neutrophil count, platelet count, hemoglobin (HB) level, and the neutrophil–lymphocyte ratio (NLR).

### 2.3. Statistical Analysis

Descriptive statistics was applied to summarize baseline characteristics, tumor features, and postoperative outcomes. Continuous variables were compared using Student’s *t*-test or Mann–Whitney U tests, depending on the normality of distribution. Categorical variables were analyzed using Chi-square tests. Statistical significance was set at *p* under 0.05. All analyses were performed using Microsoft Excel version 16.92 and SPSS 22 (IBM Corp., Armonk, NY, USA). The results were presented with 95% confidence intervals where applicable.

### 2.4. Informed Consent

Formal consent was routinely obtained from all patients at hospital admission. As a result, no additional consents were required for this study. This study was conducted in accordance with the Declaration of Helsinki, and the protocol was approved by the Institutional Ethics Committee of the Bihor County Emergency Hospital.

## 3. Results

### 3.1. Patient Demographics and Clinical Characteristics

A total of 191 patients were enrolled in this study. Of these, 78 (40.84%) were female and 113 (59.16%) were male (Table 1). Males had a higher frequency of non-mucinous adenocarcinoma compared to females (Table 2). The average age of the sample was 69.82 ± 10.18 years, ranging from 38 to 90 years (Table 3).

### 3.2. Hematological Parameters

Summaries of hematocrit, hemoglobin, white blood cell counts, and other hematological values are presented in Table 3. The mean hemoglobin was 11.37 ± 3.81 g/dL and the mean platelet count was 353.46 ± 142.93 × 10^3^/µL.

### 3.3. Distribution of Tumor Types

Out of a total of 191 cases, adenocarcinoma (ADK) accounted for 187 cases (97.91%) while 4 cases (2.09%) were classified as carcinoma (Table 4). Within the ADK group, 36 cases (18.85%) were mucinous adenocarcinoma, while 151 cases (78.53%) belonged to other subtypes (Table 4).

An independent *t*-test was performed to compare MAC to non-MAC across hematological parameters (WBC, LYM, NEU, MONO, PLT, HGB, and HCT). All *p*-values are greater than 0.05, showing no statistical differences between the two groups. Confidence intervals for each parameter included zero, reinforcing the lack of significant difference. Table 5 provides details on *p*-values and confidence intervals.

In summary, these findings suggest there is no statistically significant difference between mucinous ADK and non-mucinous types of colon cancer regarding the hematological parameters.

A one-way ANOVA test was conducted to examine the differences among various histopathological subtypes. It revealed significant differences for three parameters, WBC, NEU, and MONO. The details are presented in Table 6.

The Tukey post hoc test results for WBC show several significant and non-significant differences between group pairs. The results emphasize that the mucinous adenocarcinoma group shows a significant difference when compared to the anaplastic group. The test results for neutrophils show several significant and non-significant differences between group pairs, showing that the mucinous adenocarcinoma group shows a significant difference when compared to the anaplastic group. The test results for monocytes highlight significant differences in monocyte levels between several group pairs, notably between mucinous ADK and mixed MiNEN type, with mucinous showing a significantly lower difference. Table 7 details the specific pairwise comparisons and adjusted *p*-values.

**Figure 1:**
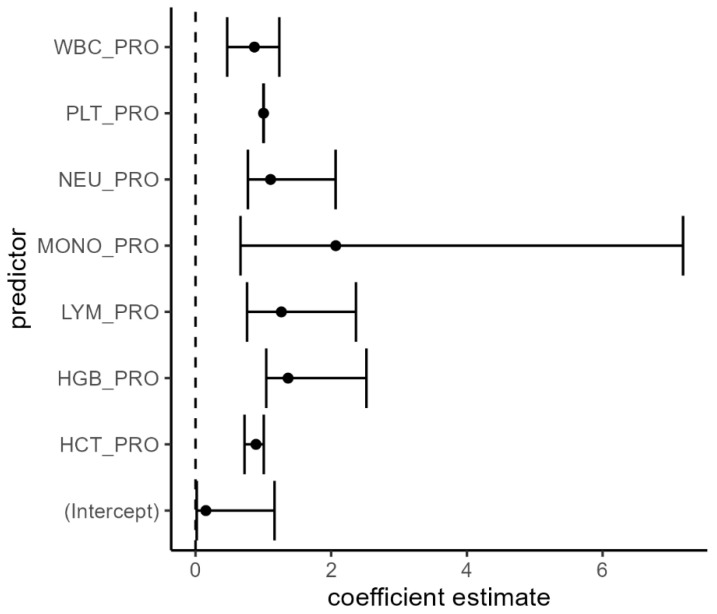
The predictors vs the coefficient estimate. The coefficient estimates for each predictor from both simple and multiple logistic regression models.

Simple logistic regressions were fitted to predict the odds of finding mucinous adenocarcinoma based on hematological parameters. While the model’s intercepts were significant, indicating that the model’s base level (without considering the predictors) significantly affects the outcome, none of the predictors (WBC, LYM, NEU, MONO, PLT, HGB, and HCT) reached statistical significance. This suggests that these blood parameters alone do not predict the mucinous type in the model tested (Figure 1). Table 8 presents the results of the logistic regression analysis.

The results from the multiple logistic regression shown in Table 9, reveal that the intercept was not significant (*p* = 0.08), and none of the predictors (WBC, LYM, NEU, MONO, PLT, HGB, and HCT) showed statistical significance, as their *p*-values were all above 0.05. This indicates that none of these blood parameters significantly predict the likelihood of having mucinous adenocarcinoma versus the other types of cancer (Figure 1).

The key characteristics of the independent variables related to emergency admission, diagnostic findings, surgical procedures, complications, and outcomes for 191 patients with colon cancer are summarized in Table 10. These findings provide a comprehensive overview of the surgical and diagnostic landscape for colon cancer cases that are presented to the hospital, highlighting the critical areas of patient management and outcomes.

Bowel obstruction (68.8%) was the most common acute presentation, followed by peritonitis. Tumoral perforation was the leading cause of peritonitis (45.9%). The sigmoid colon was the most frequent tumor site (49.2%).

Most surgeries were segmental resections (50.26%). Anastomoses were performed in 48.2% of cases. The overall complication rate was 13.1%, most often involving evisceration or anastomotic fistula, and 13.1% of the patients required at least one reintervention.

In-hospital mortality was 18.8%. Regarding metastasis, 18.3% had liver metastasis (most commonly affecting both lobes) and pulmonary metastasis and peritoneal metastasis were observed in 3.7% and 5.2% of patients, respectively.

The frequency distribution of surgical interventions for mucinous adenocarcinoma versus non-mucinous colon cancer highlights some key patterns that are presented in Table 11. Regarding the presentation as elective or as an emergency, patients with mucinous adenocarcinoma had 58.3% emergency presentations, compared to the non-mucinous types that presented as an emergency in 67.2% of cases. The predominant emergency diagnostic was bowel obstruction for both mucinous (66.6%) and non-mucinous (69.23%) cases. However, peritonitis was slightly more common in mucinous adenocarcinoma compared to non-mucinous cases. Tumoral perforation was the main cause of peritonitis in mucinous adenocarcinoma (71.6%), compared to non-mucinous cases where intratumoral abscess and tumoral perforation were recorded as principal causes.

Regarding the tumor site, the most frequent for both types is the sigmoid colon, with 38.9% of mucinous cases and 51.6% of non-mucinous cases. In mucinous adenocarcinoma, there was a notable prevalence of tumors in the caecum (27.8%) compared to only 10.9% for non-mucinous cases. Right colectomy was more common in mucinous (50%) versus non-mucinous (25.8%) cases. Anastomosis rates were higher in non-mucinous (50.3%) than in mucinous (38.9%) cases.

CT scans and endoscopies were common diagnostic tools used in most cases, regardless of cancer type.

Surgical complications were slightly less frequent in mucinous adenocarcinoma compared to non-mucinous cases (8.3% vs. 14.2%). The in-hospital mortality was slightly higher for non-mucinous colon cancer (19.4%) compared to mucinous cases (16.7%).

Regarding metastasis, pulmonary metastasis was more common in mucinous adenocarcinoma (8.3%), and the same was found for peritoneal metastasis (8.3%) and local invasion (30.6%). Liver metastases were slightly higher in mucinous (19.4%) compared to non-mucinous (18.1%) cases, and bilateral liver involvement was more common in mucinous cases (85.7% vs. 57.1%)

The analysis reveals that mucinous adenocarcinoma is associated with more aggressive tumors, with higher rates of metastasis (Table 11).

A Chi-square analysis was conducted to assess the association between mucinous adenocarcinoma of the colon and various clinical or diagnostic factors that are presented in Table 12.

The Chi-square analysis revealed several significant and non-significant associations with various clinical and diagnostic parameters. Most comparisons were non-significant, indicating that mucinous adenocarcinoma did not substantially influence emergency presentation, diagnostic findings, tumor location, surgical complications and reinterventions, or distant metastases.

The type of surgery performed showed a significant relationship with mucinous adenocarcinoma (X^2^ = 14.5223; *p*-value = 0.0126). Patients with mucinous adenocarcinoma more frequently underwent right colectomy and colostomy/ileostomy compared to those with non-mucinous cancer types. This may reflect a higher prevalence of mucinous lesions in the right colon and the potential complexity of these cases.

Furthermore, a significant association was found between mucinous adenocarcinoma and local invasion (X^2^ = 4.3874; *p*-value = 0.0362), implying that this type of cancer is more prone to local invasive growth than other subtypes.

Additionally, there was a significant correlation between mucinous adenocarcinoma and general mortality (X^2^ = 4.2111; *p*-value = 0.0402), indicating that patients with mucinous adenocarcinoma have a higher mortality rate.

Overall, these findings underscore the distinct clinical profile of mucinous adenocarcinoma. The increased frequency of right-sided resections, higher mortality, and greater propensity for local invasion emphasize the need for tailored therapeutic strategies and vigilant management in patients with this subtype (Table 12).

**Figure 2:**
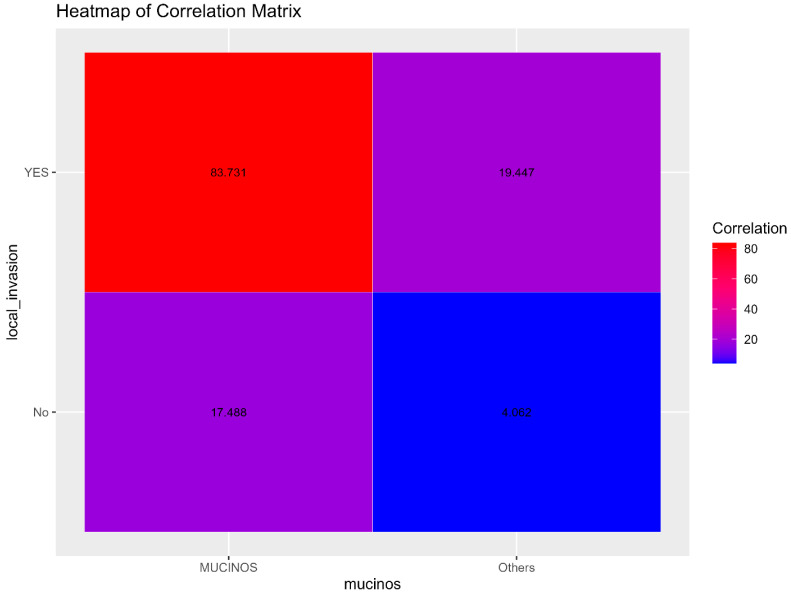
Heatmap of the correlation matrix for local invasion between the two groups. Red suggests a stronger correlation; blue suggests a weaker or no correlation.

**Figure 3:**
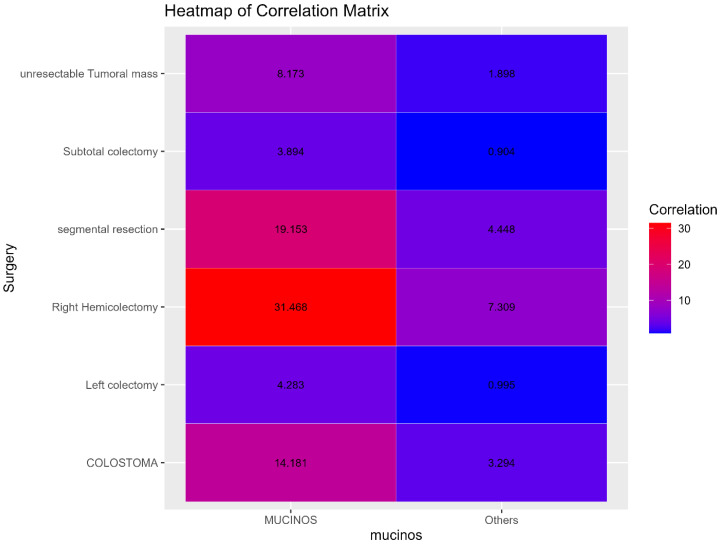
Heatmap of the correlation matrix for surgery type between the two groups.

The correlation heatmaps for local invasion and for type of surgery are presented in Figure 2 and Figure 3.

The correlation between heatmaps for local invasion and surgery type (Figure 2 and Figure 3) further illustrates these significant associations. Red areas in the heatmaps suggest stronger correlations, whereas blue areas indicate weaker or no correlation.

The average hospital stay for patients with colon cancer is 12.16 days, with a mean standard error (SE) of 0.65 days. This indicates that, on average, patients spend just over 12 days in the hospital, with a relatively small variation around this mean, suggesting a consistent length of stay across the patient population studied.

As shown in Table 13, the distribution of the length of hospital stay for patients with adenocarcinoma shows a wide range of values. The minimum length of stay was 1 day, while the maximum reached up to 78 days. The data are summarized by the following key statistics: 5th percentile: 2.5 days; 10th percentile: 5 days; 25th percentile (Q1): 7 days; median (50th percentile): 10 days; 75th percentile (Q3): 14 days; 90th percentile: 22 days; and 95th percentile: 27 days.

These percentiles indicate that most patients have a length of stay between 7 and 14 days, with a notable increase in time observed at the higher percentiles, reflecting some instances of prolonged hospitalization.

When comparing the LOS between the mucinous adenocarcinoma and non-mucinous cancer types (Table 14), the following results were obtained:

For patients with mucinous adenocarcinoma:

The mean length of hospital stay is 10.53 days, with a standard deviation (SD) of 5.89 days. The 25th percentile (Q1) is 7.75 days, the median (50th percentile) is 9 days, and the 75th percentile (Q3) is 12 days.

For patients with non-mucinous colon cancer:

The mean length of hospital stay is 12.54 days, with a standard deviation (SD) of 9.50 days. The 25th percentile (Q1) is 7 days, the median (50th percentile) is 10 days, and the 75th percentile (Q3) is 15 days.

These statistics indicate that, on average, patients with non-mucinous colon cancer tend to have a slightly longer hospital stay compared to those with mucinous adenocarcinoma. The variability in hospital stay was also higher for patients with non-mucinous colon cancer, as indicated by the larger standard deviation.

These findings underscore the importance of considering histopathological subtype in planning and managing inpatient care for patients with colon cancer.

An independent *t*-test was conducted to compare the length of hospital stay (LOS) between patients with mucinous adenocarcinoma and those with a non-mucinous type of colon cancer. The results indicated that the mean length of hospital stay for patients with mucinous adenocarcinoma (*n* = 36) was compared with patients with a non-mucinous type of colon cancer (*n* = 155). The t-statistic was −1.61 with 83.09 degrees of freedom, yielding a *p*-value of 0.109. This result is not statistically significant, indicating that there is no significant difference in the length of hospital stay between the two groups.

**Figure 4:**
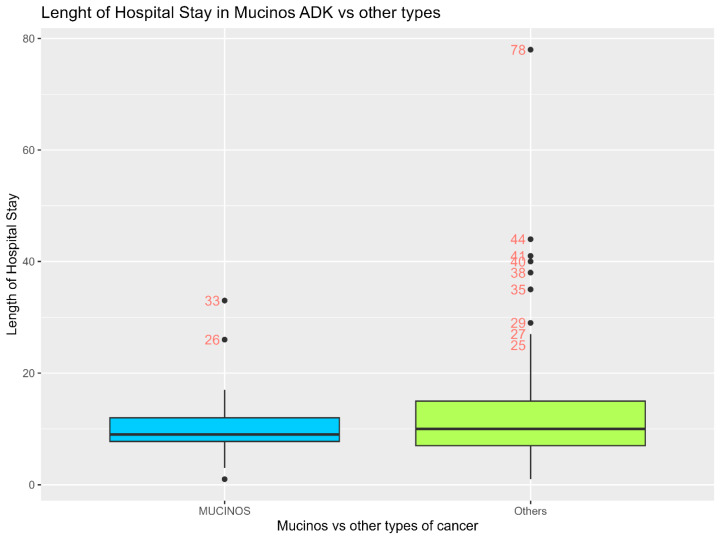
Boxplot of the length of hospital stay in the two groups.

Figure 4 displays a boxplot illustrating the LOS in both groups. Mucinous adenocarcinoma cases exhibit a more consistent length of stay with fewer extreme outliers, while other types of cancer show greater variability and more extreme outliers. This aligns with the observation that the mean length of stay difference between the groups is not statistically significant.

## 4. Discussion

This study demonstrates that mucinous adenocarcinoma of the colon is associated with distinct clinical outcomes, including a higher prevalence of local invasion and a trend toward increased overall mortality. However, no significant differences in hematological parameters were observed between MAC and non-MAC cases.

Mucinous adenocarcinoma of the colon is a distinct subset of colon cancer characterized by significant mucin production, which can impact both biological behavior and clinical outcomes. Compared to non-mucinous types of colon cancer, mucinous adenocarcinomas often exhibit increased resistance to conventional chemotherapy, present at more advanced stages, and show a less favorable prognosis. The differential expression of molecular markers and signaling pathways between mucinous and non-mucinous cancers offer crucial insights into the tumorigenic processes involved and highlight the need for specific diagnostic and treatment strategies. Thus, understanding these differences is essential for enhancing patient management.

In the Surveillance, Epidemiology, and End Results Program (SEER) conducted between 1975 and 2016, mucinous ADK of the colon presented nearly 39% of all solid tumors by body regions while solid, non-blood borne, non-mucinous tumors represent only 7% of colon cancers; mucinous ADK of the colon cases presented only 0.7% of all regional mucinous ADK cases [14,15].

### 4.1. Demographics and Overall Prevalence

In the present study of 191 patients, males constituted the majority of cases, yet the frequency of mucinous adenocarcinoma was equally distributed between sexes. The mean patient age approached 70 years, in line with previous research indicating that it predominantly affects older adults [16,17,18,19,20,21]. The broad age range (38–90 years) underscores that both mucinous and non-mucinous colon cancers can manifest across a wide spectrum of patient ages. Studies show that males have a 4.22 times higher risk of death compared to females [20]. A recent study suggests higher frequencies of mucinous ADK in patients over 65 years and in females [15].

### 4.2. Hematological Parameters

The hematological parameters were evaluated across mucinous and non-mucinous tumors, including white blood cells (WBCs), lymphocytes (LYMs), neutrophils (NEUs), monocytes (MONO), platelets (PLTs), hemoglobin (HGB), and hematocrit (HCT). While there were some statistically significant differences in WBC, NEU, and MONO counts, none of these metrics served as reliable predictors of clinical outcomes in this cohort. This aligns with existing studies suggesting that, although changes in inflammatory markers may reflect systemic disease processes, they do not independently predict survival or disease progression.

Studies have shown that iron-deficiency anemia is an independent predictor of long-term outcomes in advanced cancer [22], anemia being more frequently associated with right-sided tumors, visible blood in stools, and changes in bowel habits [23]. Some studies show that hemoglobin levels are significant predictors of disease-free survival [24]. Elevated neutrophil and monocyte count in peripheral blood are linked to poor prognosis for overall and disease-free survival, while lower levels of lymphocytes, eosinophils, red blood cells, and hemoglobin are associated with postoperative disease progression and death [15]. Anemia is also a notable clinical feature of mucinous adenocarcinoma [25]. Additionally, a case report identified low hemoglobin and hematocrit with macrocytosis indicative of Fanconi anemia in a bone-transplanted patient with mucinous adenocarcinoma [26,27].

However, none of the hematological parameters in our study demonstrated predictive value, which is consistent with the understanding that while these changes are indicative of the disease, they do not independently predict outcomes [20,24].

### 4.3. Tumor Location and Surgical Intervention

In line with prior findings [15,16,17,19,21], our data showed that mucinous adenocarcinomas were predominantly located in the right colon. Our results indicated that mucinous adenocarcinoma was frequently located in the caecum and ascending colon, although the sigmoid colon remained a major site as well.

It is statistically more frequent in the right colon compared to non-mucinous ADK [17,19,21].

Furthermore, mucinous cases showed a modestly increased use of colostomy, suggesting higher operative complexity.

### 4.4. Complications and Mortality

Patients with non-mucinous colon cancer underwent emergency surgeries more frequently, often due to bowel obstruction. Segmental resections were commonly performed in these cases. Most patients did not experience complications and did not require reinterventions.

Studies have shown that mucinous ADK patients tend to have a lower rate of curative colonic resection compared to non-mucinous ADK patients [18]. Postoperative complications are presented in the literature as follows: 74.2% of patients experience no or minor complications (Clavien–Dindo grade 1 or 2) and 25.8% experience major complications (Clavien–Dindo grade ≥ 3) [28]. The most frequent postoperative complication is surgery-related infections, affecting 23.6% of patients, and postoperative anastomotic leakage, occurring in 10.4% of cases [29]. Anastomosis leakage is a significant early complication that increases the risk of infection and peritonitis [30]. These complications are not correlated with the age of the patient, but postoperative reintervention and emergency surgery are independent risk factors for 30-day mortality [28]. Additionally, local recurrence and distant metastasis occur in 23% and 20% of mucinous ADK patients, respectively [31,32]. Bowel obstruction and infiltrative growth type are identified as independent prognostic factors [20].

Our data showed a significant correlation between mucinous adenocarcinoma and local tumor invasion (*p* < 0.5), reflecting the aggressive local behavior often reported in the literature [18,20]. The overall surgical complication rate did not differ between the subtypes.

Mucinous adenocarcinoma (ADK) of the colon is associated with poorer survival rates compared to non-mucinous ADK and carcinoma patients. Specifically, mucinous ADK patients exhibit lower survival rates irrespective of distribution [16], with a lower survival rate than non-mucinous colon cancer patients [18]. The five-year survival rate is also lower for mucinous ADK [20]. After a follow-up period of 65 months, 83% of patients with progressive disease died [21]. A meta-analysis revealed that mucinous ADK patients have a 1.4 times higher risk of death compared to non-mucinous ADK [33], and a population-based study confirmed lower five-year survival for mucinous ADK patients [15]. However, right-sided mucinous ADK showed a better survival rate than left-sided [34].

According to the findings of our study, although in-hospital mortality rates were comparable between the two groups, we identified a significant relationship between mucinous adenocarcinoma and overall mortality. This finding agrees with multiple reports, indicating poorer long-term survival among mucinous adenocarcinoma patients [16,18,20,33].

The distinct biology of mucinous tumors, potentially involving advanced local invasion, complex surgical management, and late-stage presentation, could explain this elevated mortality risk.

Therefore, consistent with the literature, mucinous ADK has the worst prognosis compared to the non-mucinous type.

### 4.5. Length of Hospital Stay

The length of hospital stay (LOS) after colorectal surgery varies depending on the type of procedure and patient characteristics. Laparoscopic colorectal surgery is associated with the lowest complication rates and shorter hospital stays [35]. Patients with mucinous colorectal adenocarcinoma experience a shorter LOS after laparoscopic surgery compared to open surgery, although the 3-year and 5-year disease-free survival rates are statistically insignificant [31,32]. The postoperative hospital stay after hepatic resection for colorectal cancer liver metastasis is approximately 11 days [36]. For patients undergoing right-sided colon cancer surgery, the average LOS is 14 days [37]. Furthermore, the median hospital stay for colorectal cancer patients is estimated at 13 days, with a maximum of 164 days observed in patients over 65 years old [28].

Our study indicates that, on average, patients spend just over 12 days in the hospital, with a relatively small variation around this mean, suggesting a consistent length of stay across the patient population studied. The minimum length of stay was 1 day, while the maximum was 78 days. These findings are in alignment with the existing literature.

### 4.6. Clinical Implications

These results emphasize the complexity of mucinous adenocarcinoma management. Mucinous lesions are associated with heightened local invasion, unique surgical challenges, and higher overall mortality risk. These features suggest the need for more vigilant surgical planning, comprehensive oncological treatment protocols, and close patient monitoring.

### 4.7. Limitations

This study has several limitations that should be considered when interpreting the results:

- The retrospective design.
- Confounding bias: while comparisons were made between mucinous and non-mucinous cancer, potential confounding factors such as age, sex, comorbidities, and other demographic or clinical variables were not fully adjusted for statistical analysis.
- This study was conducted at a single institution, which may limit the generalizability of the findings to other populations or healthcare settings.
- The relatively small number in the mucinous adenocarcinoma group may have limited the statistical power to detect differences in certain outcomes, such as surgical complications.
- Potentially relevant variables, such as genetic or molecular markers, were not included in the analysis. These factors should be considered in future research.
- This study focused primarily on short-term clinical and postoperative outcomes. Recurrence rates and quality of life measures were not assessed.

Despite these limitations, this study provides valuable insights into the differences between mucinous adenocarcinoma and non-mucinous colon cancer, emphasizing the need for further research to expand upon these findings.

## 5. Conclusions

This study highlights the importance of recognizing mucinous adenocarcinoma of the colon as a distinct pathological entity. While immediate postoperative outcomes (such as length of stay and complication rates) may be broadly similar to the non-mucinous types, the higher mortality and tendency toward local invasion demand careful multidisciplinary management and follow-up.

In summary, this study highlights the distinct clinical and surgical profiles of mucinous and non-mucinous adenocarcinomas of the colon. The unique challenges associated with the MAC type, such as the aggressive local invasion and high mortality, underscore the necessity for careful treatment approaches.

This study’s limited sample size necessitates larger multicenter research to validate the findings and explore additional prognostic factors. Future studies should focus on identifying molecular biomarkers or therapeutic targets that could refine risk stratification and guide the treatment.

Overall, our results align with the existing literature and emphasize the necessity for tailored medical and surgical strategies to address the distinct risks associated with each type of cancer. The management of mucinous adenocarcinoma requires careful consideration of its unique characteristics and higher mortality risk. These findings underscore the importance of personalized treatment plans to improve patient outcomes in colorectal cancer.

## Figures and Tables

**Table 1:** The frequency table of sex distribution in patients enrolled in this study.

	Freq	% Valid	% Valid Cum.	% Total	% Total Cum.
Female	78	40.84	40.84	40.84	40.84
Male	113	59.16	100	59.16	100
Total	191	100	100	100	100

**Table 2:** Distribution of sex and mucinous adenocarcinoma.

	Mucinous	Non-Mucinous
Female	18 (9.4%)	60 (31.4%)
Male	18 (9.4%)	95 (49.7%)

**Table 3:** Descriptive statistics of the hematological profile.

	Mean	SD	Min	Q1	Median	Q3	Max	IQR	CV
Age	69.82	10.18	38	64	69	77	90	13	0.15
HCT	37.87	29.57	12.6	29.47	36.23	42.09	42.6	12.53	0.78
HGB	11.37	3	3.81	9.41	11.58	13.83	17.36	4.41	0.26
LYM	1.63	1.15	0.19	0.9	1.45	1.93	9.54	1.02	0.71
MONO	0.73	0.44	0.1	0.43	0.62	0.87	2.63	0.44	0.61
NEU	9.56	6.95	1.59	5.11	7.78	11.19	49.19	6.07	0.73
PLT	353.46	142.93	62	245	335	428	793	183	0.4
WBC	12.14	7.41	1.91	7.4	10.3	14.6	53.17	7.11	0.61

SD: standard deviation, min: minimum, max: maximum, Q1: first quartile, Q3: third quartile, and CV: coefficient of variance.

**Table 4:** Frequency distribution of the histopathological type.

	Freq	% Valid	% Valid Cum.	% Total	% Total Cum.
Adenocarcinoma	187	97.91	97.91	97.91	97.91
Carcinoma	4	2.09	100	2.09	100
Total	191	100	100	100	100
NOS	150	78.53	78.53	78.53	78.53
Mucinous	36	18.85	97.38	18.85	97.38
Cribriform	2	1.05	98.43	1.05	98.43
Anaplastic	1	0.52	98.95	0.52	98.95
Mixed (neuroendocrine–non-neuroendocrine)	1	0.52	99.48	0.52	99.48
Multifocal	1	0.52	100	0.52	100
Total	191	100	100	100	100

**Table 5:** Independent *t*-test results for mucinous adenocarcinoma versus other types of colon cancer for hematological parameters.

Parameter	*p*-Value	CI Lower	CI Upper	Significance
WBC	0.86	−2.13	2.54	NS
LYM	0.7	−0.66	0.45	NS
NEU	0.77	−1.89	2.55	NS
MONO	0.34	−0.23	0.08	NS
PLT	0.06	−93.74	1.5	NS
HGB	0.59	−0.86	1.5	NS
HCT	0.21	−2.19	9.84	NS

NS: non-significant. CI: confidence interval.

**Table 6:** Hematological profile.

ITEM	F-Statistics	*p*-Value	Significance
White blood cell	7.36	2.59020200536523 × 10^−6^	Significant
Lymphocyte	0.57	0.723793541631166	Not significant
Neutrophils	7.8	1.11239506604841 × 10^−6^	Significant
Monocyte	5.89	4.46768381718019 × 10^−5^	Significant
Platelet	1.05	0.388772831862533	Not significant
Hemoglobin	0.82	0.537894828766022	Not significant
Hematocrit	0.15	0.980563700299152	Not significant

**Table 7:** The Tukey post hoc test results for WBC, NEU, and MONO.

	WBC		NEU		MONO	
	*p*-Adj	Significance	*p*-Adj	Significance	*p*-Adj	Significance
Cribriform–anaplastic	0	Significant	0	Significant	0.03	Significant
Mixed MiNEN (neuroendocrine–non neuroendocrine)–anaplastic	0	Significant	0	Significant	0.95	Not significant
Mucinous–anaplastic	0	Significant	0	Significant	0.08	Not significant
Multifocal–anaplastic	0	Significant	0	Significant	0.09	Not significant
NOS–anaplastic	0	Significant	0	Significant	0.04	Significant
Mixed MiNEN (neuroendocrine–non neuroendocrine) cribriform	0.99	Not significant	1	Not significant	0	Significant
Mucinous cribriform	1	Not significant	1	Not significant	0.66	Not significant
Multifocal cribriform	1	Not significant	1	Not significant	1	Not significant
NOS cribriform	1	Not significant	1	Not significant	0.82	Not significant
Mucinous mixed MiNEN (neuroendocrine–non-neuroendocrine)	1	Not significant	1	Not significant	0	Significant
Multifocal mixed MiNEN (neuroendocrine–non-neuroendocrine)	0.97	Not significant	1	Not significant	0.01	Significant
NOS mixed MiNEN (neuroendocrine–non-neuroendocrine)	1	Not significant	1	Not significant	0	Significant
Multifocal mucinous	0.99	Not significant	0.99	Not significant	0.9	Not significant
NOS mucinous	1	Not significant	1	Not significant	0.87	Not significant
NOS multifocal	0.99	Not significant	0.99	Not significant	0.96	Not significant

**Table 8:** Logistic regression of mucinous ADK vs hematological parameters—odds ratios.

Term	Estimate	Std. Error	Statistic	Conf. Low	Conf. High	*p*-Value	Significance
(Intercept)	0.24	0.36	−3.94	0.12	0.5	0	Significant
WBC	1	0.03	−0.15	0.94	1.04	0.88	Not significant
(Intercept)	0.21	0.31	−5.05	0.11	0.38	0	Significant
LYM	1.08	0.15	0.49	0.78	1.43	0.62	Not significant
(Intercept)	0.25	0.32	−4.37	0.13	0.47	0	Significant
NEU	0.99	0.03	−0.25	0.93	1.04	0.8	Not significant
(Intercept)	0.18	0.35	−4.89	0.09	0.35	0	Significant
MONO	1.43	0.39	0.92	0.64	3.03	0.36	Not significant
(Intercept)	0.11	0.51	−4.43	0.04	0.28	0	Significant
PLT	1	0	1.73	1	1	0.08	Not significant
(Intercept)	0.35	0.71	−1.49	0.08	1.35	0.14	Not significant
HGB	0.96	0.06	−0.58	0.86	1.09	0.56	Not significant
(Intercept)	0.42	0.75	−1.15	0.16	1.82	0.25	Not significant
HCT	0.98	0.02	−0.81	0.94	1.01	0.42	Not significant

**Table 9:** Multiple logistic regression of mucinous ADK vs hematological parameters–odds ratios.

Term	Estimate	Std. Error	Statistic	Conf. Low	Conf. High	*p*-Value	Significance
(Intercept)	0.15	1.06	−1.77	0.02	1.16	0.08	Not significant
WBC	0.87	0.27	−0.52	0.47	1.24	0.6	Not significant
LYM	1.27	0.3	0.79	0.76	2.36	0.43	Not significant
NEU	1.11	0.27	0.37	0.77	2.07	0.71	Not significant
MONO	2.07	0.6	1.21	0.66	7.19	0.23	Not significant
PLT	1	0	1.66	1	1	0.1	Not significant
HGB	1.36	0.3	1.04	1.04	2.52	0.3	Not significant
HCT	0.89	0.1	−1.13	0.72	1.01	0.26	Not significant

**Table 10:** Summary of frequency distribution of surgical intervention and complications.

	All Cases	Freq	Percentage
Emergency	No	66	34.6%
	Yes	125	65.4%
	Total	191	100%
Emergency diagnostics	Abdominal wall abscess	2	1.6%
	Bowel obstruction	86	68.8%
	Fecaloid peritonitis	21	16.8
	Purulent peritonitis	16	12.8%
	Total	125	100%
Cause of peritonitis	Colo-vesical fistula	1	2.7%
	Diastatic perforation	7	18.9%
	Intratumoral abscess	12	32.4%
	Tumoral perforation	17	45.9%
	Total	37	100%
Localization of the tumoral process	Ascending colon	23	12%
	Caecum	27	14.1%
	Descending colon	16	8.4%
	Sigmoid colon	94	49.2%
	Transverse colon	31	16.2%
	Total	191	100%
Surgery	Stomy without resection	9	4.71%
	Left colectomy	25	13.08%
	Right colectomy	58	30.36%
	Segmental resection	96	50.26%
	Subtotal colectomy	3	1.57%
	Total	191	100%
Bowel restoration	Anastomosis	92	48.2%
	Stoma	98	51.8%
	Total	191	100%
Anastomosis type	L-L	14	15.21%
	L-T	1	1.08%
	T-L	9	9.78%
	T-T	68	73.91%
	Total	92	100%
Surgical complications	No	166	86.9%
	Yes	25	13.1%
	Total	191	100%
Complication	Anastomotic fistula	6	24%
	Colon necrosis	1	4%
	Diastatic fistula	3	12%
	Evisceration	12	48%
	Fournier gangrene	1	4%
	Hematoma	1	4%
	Spleen necrosis	1	4%
	Total	25	100%
Reinterventionsurgery needed	No reintervention	166	86.91%
	Yes	25	13.1%
	Total	191	100%
Third intervention needed	No	188	98.4%
	Yes	3	1.6%
	Total	191	100%
In-hospital death	No	155	81.2%
	Yes	36	18.8%
	Total	191	100%
CT scan	No	39	20.4%
	Yes	152	79.6%
	Total	191	100%
Endoscopy	No	108	56.5%
	Yes	83	43.5%
	Total	191	100%
Liver metastasis	No	156	81.7%
	Yes	35	18.3%
	Total	191	100%
Liver lobes affected	Both	22	62.85%
	Left	1	2.85%
	Right	12	34.28%
	Total	35	100%
Pulmonary metastasis	No	184	96.3%
	Yes	7	3.7%
	Total	191	100%
Peritoneal metastasis	No	181	94.8%
	Yes	10	5.2%
	Total	191	100%
Local invasion	No	158	82.7%
	Yes	33	17.3%
	Total	191	100%
Histopathologic exam	Biopsy	9	4.7%
	Specimen	182	95.3%
	Total	191	100%
Histopathological type	Mucinous	36	18.8%
	Others	155	81.2%
	Total	191	100%

**Table 11:** Frequency distribution of demographic data, surgical details, and histopathological findings in mucinous compared to non-mucinous carcinomas.

Category	Item	Mucinous (*n* = 36)	% Mucinous	Others (*n* = 155)	% Others	Total Cases (*n* = 191)
Emergency	No	15	41.7%	51	32.9%	66
	Yes	21	58.3%	104	67.1%	125
Emergency diagnostics (mucinous *n* = 21, others *n* = 104)	Abdominal wall abscess	0	0%	2	1.9%	2
	Bowel obstruction	14	66.6%	72	69.2%	86
	Fecaloid peritonitis	4	19%	17	16.3%	21
	Purulent peritonitis	3	14.28%	13	12.5%	16
Cause of peritonitis (mucinous *n* = 7, others *n* = 30)	Colo-vesical fistula	1	14.2%	0	0%	1
	Diastatic perforation	1	14.2%	6	20%	7
	Intratumoral abscess	0	0%	12	40%	12
	Tumoral perforation	5	71.6%	12	40%	17
Localization of the tumor	Ascending colon	5	13.9%	18	11.6%	23
	Caecum	10	27.8%	17	10.9%	27
	Descending colon	1	2.8%	15	9.6%	16
	Sigmoid colon	14	38.9%	80	51.6%	94
	Transverse colon	6	16.7%	25	16.1%	31
Surgery type	Colostomy	4	11.1%	5	3.2%	9
	Left colectomy	3	8.3%	22	14.2%	25
	Right colectomy	18	50%	40	25.8%	58
	Segmental resection	11	30.6%	85	54.8%	96
	Subtotal colectomy	0	0%	3	1.9%	3
Bowel restoration	Anastomosis	14	38.9%	78	50.3%	92
	Anastomosis without resection	1	2.8%	0	0%	1
	Stomy	21	58.3%	77	49.67%	98
Anastomosis type (mucinous *n* = 15; others *n* = 78)	L-L	3	20%	12	15.38%	15
	L-T	0	0%	1	1.28%	1
	T-L	2	13.3%	7	8.97%	9
	T-T	10	66.6%	58	74.3%	68
Surgical complications	No	33	91.7%	133	85.8%	166
	Yes	3	8.3%	22	14.2%	25
Complication (mucinous *n* = 3, others *n* = 22)	Anastomotic fistula	1	33.3%	5	22.7%	6
	Colon necrosis	0	0%	1	4.5%	1
	Diastatic fistula	0	0%	3	13.6%	3
	Evisceration	2	66.6%	10	45.4%	12
	Fournier gangrene	0	0%	1	4.5%	1
	Hematoma	0	0%	1	4.5%	1
	Spleen necrosis	0	0%	1	4.5%	1
Reintervention	No reintervention	33	91.7%	133	85.8%	166
	Yes	3	8.3%	22	14.2%	25
Third reintervention	No	35	97.2%	153	98.7%	188
	Yes	1	2.8%	2	1.3%	3
In-hospital death	No	30	83.3%	125	80.6%	155
	Yes	6	16.7%	30	19.4%	36
Death occurrence up to September 2023	No	13	36.1%	88	56.8%	101
	Yes	23	63.9%	67	43.2%	90
CT scan	No	8	22.2%	31	20%	39
	Yes	28	77.8%	124	80%	152
Endoscopy	No	19	52.8%	89	57.4%	108
	Yes	17	47.2%	66	42.6%	83
Liver metastasis	No	29	80.6%	127	81.9%	156
	Yes	7	19.4%	28	18.1%	35
Liver lobes (mucinous *n* = 7, others *n* = 28)	Both	6	85.7%	16	57.14%	22
	Left	0	0%	1	3.57%	1
	Right	1	14.3%	11	39.3%	12
Pulmonary metastasis	No	33	91.7%	151	97.4%	184
	Yes	3	8.3%	4	2.6%	7
Peritoneal metastasis	No	33	91.7%	148	97.4%	181
	Yes	3	8.3%	7	2.6%	10
Local invasion	No	25	69.4%	133	85.8%	158
	Yes	11	30.6%	22	14.2%	33
Histopathologic exam	Biopsy	4	11.1%	5	3.2%	9
	Specimen	32	88.9%	150	96.8%	182

**Table 12:** Mucinous adenocarcinoma vs. clinical variables—Chi-square analysis.

Item B	Test Stat	*p*-Value	Sig
Emergency	0.6424	0.422827484616062	Not significant
Emergency diagnostics	1.4856	0.829197010016814	Not significant
Cause of peritonitis	6.965	0.13775143521464	Not significant
Localization of the tumor	8.5921	0.0721440778837877	Not significant
Surgery type	14.5223	0.0126107308636955	Significant
Intestinal continuity	5.5251	0.0631315229342296	Not significant
Anastomosis type	1.5017	0.826342793566649	Not significant
Surgical complications	0.442	0.506143639900801	Not significant
Type of complication	1.7972	0.970205587595344	Not significant
Reintervention	5.1042	0.077917720144237	Not significant
Third intervention	0	0.999999999999999	Not significant
In-hospital death	0.0182	0.892625337252098	Not significant
Death occurrence up to September 2023	4.2111	0.0401601753317112	Significant
CT scan	0.0047	0.945400202167833	Not significant
Endoscopy	0.1021	0.749349870845808	Not significant
Liver metastasis	0	1	Not significant
Liver lobes	1.4446	0.695125659075304	Not significant
Pulmonary metastasis	1.3514	0.245037600147539	Not significant
Peritoneal metastasis	0.2611	0.609369789284703	Not significant
Local invasion	4.3874	0.0362062507499573	Significant
Histopathologic exam	2.4801	0.115297211545901	Not significant

**Table 13:** Median and quartile values for the length of stay.

Min	0.05	0.10	0.25	Median	0.75	0.90	0.95	Max
1	2.5	5	7	10	14	22	27	78

**Table 14:** Median and quartile values of the LOS for mucinous adenocarcinoma versus non-mucinous types.

Mucinous	Mean	SD	Quant25	Quant50	Quant75
MAC	10.53	5.89	7.75	9	12
Others	12.54	9.50	7	10	15

## Data Availability

The original contributions presented in this study are included in the article. Further inquiries can be directed to the corresponding author.
